# Short-chain fatty acids levels in human milk are not affected by holder pasteurization and high hydrostatic pressure processing

**DOI:** 10.3389/fped.2023.1120008

**Published:** 2023-09-29

**Authors:** Lucie Marousez, Farid Ichou, Philippe Lesnik, Léa Chantal Tran, Marie De Lamballerie, Frédéric Gottrand, Delphine Ley, Jean Lesage

**Affiliations:** ^1^ Inserm, CHU Lille, U1286—INFINITE—Institute for Translational Research in Inflammation, University of Lille, Lille, France; ^2^ ICAN Omics, Foundation for Innovation in Cardiometabolism and Nutrition (ICAN), Hôpital Pitié-Salpêtrière, Paris, France; ^3^ INSERM, UMR-S1166, Sorbonne Université, Paris, France; ^4^ ONIRIS CS 82225, GEPEA, UMR CNRS 6144, Nantes, France; ^5^ Division of Gastroenterology Hepatology and Nutrition, Department of Paediatrics, Jeanne de Flandre Children’s Hospital, CHU Lille, Lille, France

**Keywords:** human milk, SCFAs, high hydrostatic pressure, holder pasteurization, sterilization

## Abstract

Sterilized donor milk (DM) is frequently used for feeding preterm infants. To date, the effect of different modes of DM sterilization on short-chain fatty acids (SCFAs) remains unknown. We aimed to quantify SCFAs in DM samples after two types of milk sterilization: the Holder pasteurization (HoP) and a high hydrostatic pressure (HP) processing. Eight pooled DM samples were sterilized by HoP (62.5°C for 30 min) or processed by HP (350 MPa at 38°C). Raw DM was used as control. Six SCFAs were quantified by gas chromatography/mass spectrometry. Compared to raw milk, both HoP and HP treatment did not significantly modulate the concentration of acetate, butyrate, propionate and isovalerate in DM. Valerate and isobutyrate were undetectable in DM samples. In conclusion, both HoP and HP processing preserved milk SCFAs at their initial levels in raw human milk.

## 1. Introduction

Short-chain fatty acids (SFCAs) are products of carbohydrate fermentation by gut bacteria (1). SCFAs are mainly concentrated in the colon and some are distributed systemically after intestinal absorption (1, 2). In the adult gut, SCFAs exert numerous beneficial functions including epigenetic effects on mucosal cells, promotion of regulatory T cell responses and tolerance, stimulation of mucus secretion, epithelial barrier integrity and dendritic cell precursors (1–3). In infants, early-life exposure to SCFAs is postulated to protect against atopy and developmental programming of immune disorders (4, 5). SCFAs are found in breast milk although the origin of these metabolites remains incompletely understood (6). Indeed, milk SCFAs may likely originate from the maternal gut microbiota and then be distributed to the mammary gland via the circulation, but they may also be produced by the resident microbiota present in breast milk (6, 7). In any case, SCFAs from breast milk may reach the digestive tract of newborns and exert early digestive roles including a strengthening of the intestinal barrier and immunity.

Preterm infants have an immature intestine that expose them to a high risk of developing diseases such as necrotizing enterocolitis and sepsis (8). Human milk banks (HMBs) provide donor milk (DM) as alternative for feeding these infants when mother's own milk is insufficient. In order to ensure the microbial safety of DM, most HMBs currently sterilize human milk (HM) using the standard method of Holder pasteurization (HoP) performed by heating milk to 62.5°C for 30 min (9). However, several studies recently demonstrated that HoP reduces some nutritional compounds of DM and also degrades numerous heat-sensitive and bioactive factors such as immunoglobulins, lactoferrin, some vitamins, lysozyme, the bile salt-dependent lipase (BSSL) and several important metabolic hormones (10, 11). High hydrostatic pressure (HP) processing, a non-thermal method, has been proposed as an alternative to HoP for DM sterilization (10, 12). Recently, it was demonstrated that using a moderate HP protocol (four cycles of 5 min at pressure of 350 MPa, performed at 38°C) numerous DM factors which are degraded by HoP are preserved by HP processing (11–14).

To date, the effect of different milk sterilization methods on milk SCFAs composition has never been studied. This study aims to evaluate the effect of DM sterilization using HoP or HP processing on the milk composition of six SCFAs (acetate, butyrate, propionate, valerate, isovalerate and isobutyrate). Gas chromatography to mass spectrometry (GC/MS) was used for SCFAs quantifications.

## 2. Materials and methods

### 2.1. Milk collection and processing

**Figure 1:**
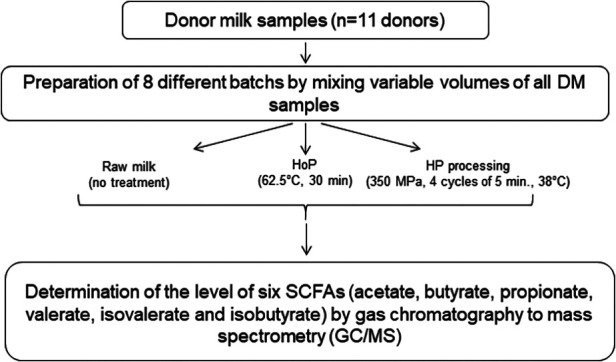
Sample collection, preparation, and the experimental design of the present study.

Frozen DM samples from 11 donors were provided by the regional HMB (Lactarium Régional de Lille, Jeanne de Flandre Children's Hospital, CHU Lille). Donors provided to our HMB written, informed consent for the use of their milk for this research purpose. Eight batches of pooled DM were created by mixing various volumes (from 10 to 30 ml) of all DM samples. Three aliquots of DM were prepared for each batch: one fraction was stored at −80°C (raw milk sample); one fraction was subjected to HoP according to the standard pasteurization protocol (62.5°C for 30 min) in the HMB of the Lille's hospital; the last fraction was subjected to HP processing as previously described (12). The set of HP parameters was as follows: pressure = 350 MPa, temperature = 38°C, number of cycles = 4 cycles of 5 min. Then, sterilized samples were stored at −80°C until analysis. Sample collection, preparation, and the experimental design of the present study are shown in Figure 1. Sample size was estimated based on previous work (11–14), common practice and G Power free software.

### 2.2. GC/MS analysis of SCFAs

Sample preparation was adapted from protocol of Zheng et al. (15). Extraction steps were carried out at 4°C. Briefly, 1 ml of human milk were used and suspended with 500 µl solution of NaOH at 0.005 M including internal standard mix of acetate-D3, butyrate-13C2, propionate-D2 and valerate-D9 at 235 µM, 88 µM, 82 µM and 41 µM, respectively. 500 µl of propanol/pyridine mix (3:2 v/v) were added to the samples and then vortexed. 50 µl of propylchloroformate (PCF) was successively added twice to the solution and vortexed. The biphasic solution was formed after addition of 300 µl of hexane and sonicated and centrifuged at 2000xg and 4°C during 5 min. 300 µl of organic phase were transferred to GC/MS vials before their injections. SCFAs were quantified by GC/MSusing an ISQ LT™ equipped with a Triplus RSH (Thermo Fisher Scientific, Illkirch, France) and a fused-silica capillary column with a (5%-phenyl)-methylpolysiloxane phase (DB-5ms, J&W Scientific, Agilent Technologies Inc., USA) of 50 m×0.25 mm i.d coated with 0.25 µm film thickness. Peaks of SCFAs were quantified using XCalibur QuanBrowser software (Thermo Fisher Scientific, Illkirch, France). Details on chromatography, calibration range, limit of detection and retention time of SCFAs are reported in supplementary materials (Supplementatary Table S1).

### 2.3. Statistical analysis

Data are presented as mean ± SEM. Statistical analysis were performed with GraphPad Prism 7.0. software (San Diego, USA). Grubb's test was used to detect any outliers. Normality of variables was evaluated by a D'Agostino-Pearson test. Statistical differences were then tested by One-way Anova (Tukey's post-test) or Kruskal-Wallis (Dunn's post-test) according to sample normality assessment results. A *p*-value <0.05 was considered significant.

## 3. Results

**Figure 2:**
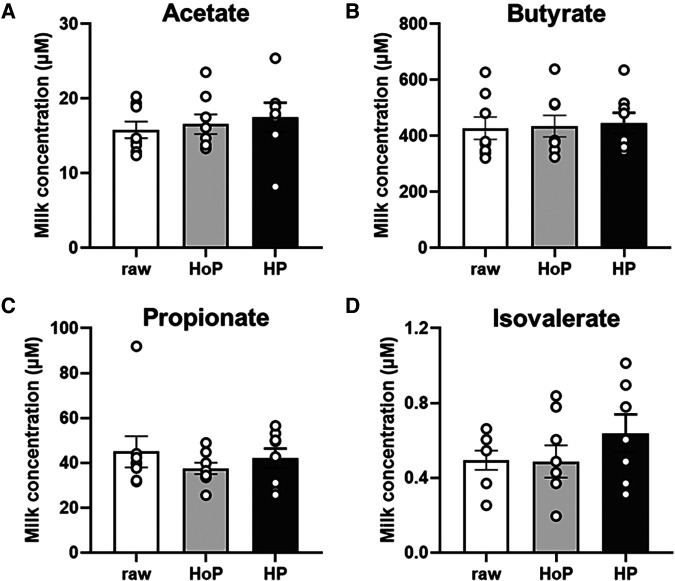
Short chain fatty acids concentrations in pooled raw donor milk, DM treated by holder pasteurization (HoP) and by high hydrostatic pressure (HP) processing. Acetate (A), butyrate (B), propionate (C) and isovalerate (D) levels. Values are expressed as mean ± SEM, *n* = 8/group (except for isovalerate and acetate in HP group, *n* = 7). Bars with SEM are the mean value in each group and dots indicate the level in individual samples.

Four SCFAs were detected at very different mean levels in raw DM: acetate 15.8 µM (range 12.4–20.3 µM) (Figure 2A); butyrate 428 µM (range 320–628 µM) (Figure 2B); propionate 45 µM (range 32–92 µM) (Figure 2C); isovalerate 0.50 µM (range 0.25–0.66 µM) (Figure 2D). Butyrate was the major SCFA detected in DM and its concentration was 9.5-fold-higher than propionate, 27-fold higher that acetate, and 856-fold-higher that isovalerate. The sterilization of DM using both HoP and HP processing did not significantly (*P* > 0.05) alter the concentration of acetate (Figure 2A), butyrate (Figure 2B), propionate (Figure 2C) and isovalerate (Figure 2D) compared to raw DM. Valerate and isobutyrate, were under the limit of detection of the GC/MS analysis (data not shown).

## 4. Discussion

The present study demonstrates that sterilization of DM with HoP or HP processing did not alter the concentration of four SCFAs present in breast milk including butyrate, propionate, isovalerate and acetate. For the first time, low levels of propionate and isovalerate were found in human milk and valerate and isobutyrate were not detected.

Values of SCFAs in breast milk are low relative to the concentration of milk medium- and long-chain fatty acids present in human milk (7, 15, 16). In accordance with other studies (16–18), butyrate was found to be the major SCFA of breast milk. For the first time, in raw and sterilized DM samples, propionate and isovalerate were detected and quantified unlike other previous studies did not detect those two SCFAs in human milk (19–21). Acetate and butyrate levels measured in the present study were similar to the concentrations and ranges reported in other studies (18–20); but, in the literature, the range of SCFAs concentrations in human milk showed great variation. Indeed, Stinson et al. (19) showed median concentrations of 46.8 µM for acetate and of 95.6 µM for butyrate in 109 DM samples at one month postpartum. Moreover, in pooled HM samples of one to two months postpartum, Prentice et al. (20) showed levels of acetate and butyrate ranging from 100 µM to 8,500 µM and from 0 to 400 µM, respectively. Paparo et al. (18), found that butyrate median concentration at 5 months postpartum was 750 µM. Conversely, a recent study only found a mean butyrate level at 144 µM at 6 months postpartum in 25 mothers who exclusively breastfed their infants for at least 3 months (22). Although the discrepancies between studies may be associated with different methods in the SCFAs quantification, those may also be due to the high inter-individual variability in SCFAs production. Indeed, levels of SCFAs in breast milk are highly variable due to maternal environmental factors, such as diet, and maternal gut microbial concentration (16–19). These differences between studies could also be due to the timing of milk sampling as SCFAs levels change during lactation (18, 22).

Present data demonstrated that both thermal sterilization of DM with HoP or using the non-thermal HP processing did not affect SCFAs concentrations. In accordance, others previous studies from others groups also demonstrated that these two types of treatment did not affect DM fatty acids levels (23, 24). However, in a recent study using an untargeted metabolomic analysis in which more than 600 compounds were analyzed (13), it was showed that both HoP and a HP processing similar to the protocol used in the present study modified several lipids which included decreased levels of free fatty acids, phospholipid metabolites, and sphingomyelins. Moreover, these decreases were more strongly noted in HP samples rather than in HoP ones (13). As lipids account for a major source of energy during the fetal/neonatal period; further studies are needed to investigate if the sterilization DM by these two methods may have nutritional and developmental consequences. Recent findings have proposed that milk SCFAs may prevent inflammation, infection and maintain gut homeostasis during development.

In HMBs, HoP is the main process used to pasteurize DM but this method, due its thermal effect, alters numerous bioactive milk compounds (9–11). More recently, the non-thermal HP processing has been investigated and even if it was demonstrated that both processes lead to the appropriate inactivation of vegetative bacterial forms in human milk, it was clearly shown that HP treatment remarkably preserves from degradation the majority of sensitive milk components (10–14). The literature (10–14) suggest that the sterilized HP-DM may be more benefic than HoP-DM for preterm newborns but, this hypothesis needs to be clinically tested. To date, the price of a Pascalizator to perform HP treatments remains high and this method is not used in HMBs so far (25). HoP leads to a high failure rate of DM microbial decontamination that is close to 10% in most HMBs (25, 26). The use of HP processing instead of HoP was shown to drastically reduce these failures (25, 26). We propose that HP processing could permit to reduce the important losses of DM each year in HMBs and that the coast of a Pascalizator can be rapidly amortized.

## 5. Conclusion

In conclusion, we demonstrated that our HP protocol [4 cycles of a moderate pressure (350 MPa) during 5 min performed at 38°C] as well as HoP preserve the main SCFAs in HM. This reinforces the potential of these two sterilization methods to treat DM in HMBs to ensure the microbial safety of DM and to preserve important milk bioactive factors including SCFAs.

## Data Availability

The raw data supporting the conclusions of this article will be made available by the authors, without undue reservation.
