# Supplementary material for: Short-chain fatty acids levels in human milk are not affected by holder pasteurization and high hydrostatic pressure processing
Source: Front Pediatr. 2023 Sep 29;11:1120008. doi: 10.3389/fped.2023.1120008 (PMC10570738; doi:10.3389/fped.2023.1120008)
Supplement: Supplementary file 1 [file Presentation1.pdf]

Supplemental material

Chemicals and Reagents. Reference and Stable isotope-labeled compounds (Acetate, propionate, butyrate, isobutyrate, valerate, isovalerate, acetate-D3, butyrate-13C2 and valerate-D9) were purchased from Sigma-Aldrich (Saint Quentin Fallavier, France). Analytical grade of NaOH, propan-1-ol, pyridine, hexane and propylchloroformate (PCF) were purchased as well from Sigma-Aldrich (Saint Quentin Fallavier, France). Deionized water comes from a Milli-Q Elix system fitted with a LC-PaK and a MilliPak filter at 0.22µm (Merck Millipore, Guyancourt, France).

GC/MS analysis of SCFAs. Sample preparation was adapted from protocol of Zheng et al. *[Zheng et al. Metabolomics. 2013 August 1; 9(4): 818–827. doi:10.1007/s11306-013-0500-6.]. Briefly, e*xtraction steps were carried out at 4°C to avoid the loss of SCFA species. 1000 µl of human breast milk were used and suspended with 500µl of a solution of NaOH at 0.005M in 5ml glass tube including internal standard mix of acetate-D3, butyrate-13C2 and valerate-D9 at 235 µM, 88µM and 41 µM and ceramic beads. Samples were homogenized at 6500 rpm, 3x20s using Prescellys® Evolution (Bertin Technologies, Montigny-le-Bretonneux, France). 500µl of propanol/pyridine mix (3:2 v/v) were added and then vortexed. 50µl of PCF was successively added twice to the solution and vortexed. The biphasic solution was The biphasic solution was formed after addition of 500µl of hexane and sonicated and centrifuged at 2000xg and 4°C during 5min. 300µl of organic phase were transferred to GC/MS vials before theirs injections. SCFAs were quantified by Gas Chromatographic/mass spectrometry using an ISQ LT™ equipped with a Triplus RSH (Thermo Fisher Scientific, Illkirch, France) and a fused-silica capillary column with a (5%-phenyl)-methylpolysiloxane phase (DB-5ms, J&W Scientific, Agilent Technologies Inc., USA) of 50m x 0.25 mm i.d coated with 0.25 µm film thickness.

Temperatures of the front inlet, MS transfer line, and electron impact ion source were set at 260°C, 290°C, and 230°C, respectively. Helium was supplied with carrier gas at a flow rate of 1 ml/min. Oven temperature was set initially at 50°C during 1.5min. Temperature was raised to 70°C at 8°C/min and to 85°C at 6°C/min. Then, temperature was successively elevated to 110°C at 22°C/min and to 120°C at 12°C/min. Oven temperature was finally set to 300°C at 125°C/min and held 3min. The run time was 15min in targeted SIM mode. Injected sample volume was set to 1µl in split mode with a 20:1 ratio. Data processing was performed using Xcalibur® software (version 3.0, Thermofisher Scientific, Illkirch, France) and details about ion transition, limit of detection, limit of quantification, calibration equation and linearity range is reported in the following table S1.

Table S1. GC/MS and quantitative parameters of SCFAs analysis in human milk. Linearity, calibration equation, recovery, limit of detection (LOD) and limit of quantification (LOQ)

SCFA species	*Mass (m/z)*	*Retention time (min)*	*Internal standards*	*Calibration equation (y=Ax+B)*	*R2*	*Linearity range (µM)*	*LOD (µM)**	*LOQ (µM)**	*Recovery (%)*
Acetate	43, 60	3.9	Acetate-D3	y = 1.125x + 0.033	0.999	1.5-700	0.44	1.48	85.9 ± 0.9
Propionate	57, 75	5.2	Propionate-D2	y = 0.680x + 0.183	0.995	1.5-600	1.03	3.42	76.5 ± 0.9
Isobutyrate	43, 71	8.5	Butyrate-13C2	y = 1.785x + 0.002	0.997	0.5-200	0.05	0.16	93.3 ± 0.8
Butyrate	43, 71	6.8	Butyrate-13C2	y = 1.019x + 0.013	0.998	1.5-1000	0.05	0.17	91.5 ± 0.7
Isovalerate	73, 85	7.4	Valerate-D9	y = 0.667x + 0.004	0.999	0.3-200	0.03	0.09	86.2 ± 0.2
Valerate	73, 85	6.0	Valerate-D9	y = 0.672x + 0.005	0.999	0.2-200	0.03	0.09	86.4 ± 1.7

**LOD=3xSD(B)/A ;LOQ=10xSD(B)/A; where SD(B) is the standard deviation of the blank and the slope (A) of the calibration curve*
